# Descriptive and Time-Series Analysis of Rabies in Different Animal Species in Mexico

**DOI:** 10.3389/fvets.2022.800735

**Published:** 2022-04-01

**Authors:** Reyna Ortega-Sánchez, Isabel Bárcenas-Reyes, Germinal Jorge Cantó-Alarcón, Jesús Luna-Cozar, Rojas-Anaya E, Yesenia G. Contreras-Magallanes, Sara González-Ruiz, Baltazar Cortez-García, Feliciano Milián-Suazo

**Affiliations:** ^1^Doctorado en Ciencias Biológicas Facultad de Ciencias Naturales, Universidad Autónoma de Querétaro, Querétaro, Mexico; ^2^Facultad de Ciencias Naturales, Universidad Autónoma de Querétaro, Querétaro, Mexico; ^3^Instituto Nacional de Investigaciones Forestales, Agrícolas y Pecuarias-Centro de Investigación Regional Pacífico Centro, Guadalajara, Mexico; ^4^Jefe de Departamento de Rabia Paralítica y Garrapata, Servicio Nacional de Sanidad, Inocuidad y Calidad Agroalimentaria (SENASICA), Ciudad de Mexico, Mexico

**Keywords:** spatio-temporal, analysis, rabies, epidemiology, Mexico

## Abstract

The spatio-temporal epidemiology of rabies has related the influence of environmental factors and anthropogenic changes on the movements of the hematophagous bat *Desmodus rotundus*. In Mexico, *D. rotundus* is the main transmitter of the rabies virus for different livestock species, modifying annually the fluctuation of the number of cases of rabies and its dissemination in subtropical areas and regions considered free of the disease. The purpose of this study was to perform a descriptive analysis of the distribution of cases of rabies in Mexico, and to perform a time-series analysis to evaluate stationarity and to predict the number of cases for the following year. A total of 3,469 cases were reported in the period of interest, of which the 89.1% occurred in cattle, 4.3% in horses, 1.5% in sheep, 0.6% in goats, 0.01% in pig, 3.1% in vampire bats, 0.3% in cervids, 0.2% in skunks, 0.1% in insectivorous bats, 0.1% in foxes, 0.1% in buffaloes, and 0.02% in coatis; 0.5% were not identified. The most frequent antigenic variants reported were AgV11, AgV5, and AgV3, associated with *D. rotundus*. The distribution of cases in bats correlates with the distribution of cases in domestic and wild animals; however, cases were observed in wild species in non-endemic areas of Mexico, like the State of Chihuahua. The additive model used in the time-series analysis showed a seasonal pattern with a peak of cases at the beginning of each year, from January to March. The model showed a good predicting value; the Pearson correlation coefficient R^2^ was 0.705. The highest probability for the occurrence of rabies cases in the different species estimated by Ordinary Kriging was in the coast of the Gulf of Mexico, involving the states of Tamaulipas, Veracruz, Tabasco, Chiapas, and Yucatan. This study confirms that rabies in domestic and wild species is endemic in tropical and subtropical areas—however, cases have been observed in new geographic areas—and provides useful information to support actions to stop the spread of the rabies virus or the reservoir, and for planning vaccination strategies considering time and place.

## Introduction

Rabies is a zoonotic disease caused by the rabies virus (RABV) from the genus *Lyssavirus*. This is a neurotrophic virus that affects all kinds of mammal species around the world (1). In humans, rabies is most often associated with dogs; however, the main reservoir for spillover to cattle is the vampire bat *Desmodus rotundus. D. rotundus* is the main transmitter of the RABV in the Americas, from the tropical areas of Mexico to the north of Argentina and Chile (2, 3). In 2019, Mexico was recognized by WHO as the first country free of dog-transmitted rabies as a public health problem (4). However, this virus still circulates in wild animals, such as vampire bats and other wildlife species. *D. rotundus* is the main transmitter of the RABV to cattle causing bovine paralytic rabies (BPR), a disease characterized by limb paralysis, neck flexion, excessive salivation, and death. The spatial–temporal epidemiology of BPR depends of the distribution of the vampire bat. Currently, in Mexico BPR is present in 25 out of 32 States, in the Pacific coast from the south of Sonora to the State of Chiapas, and in the Gulf of Mexico from the south of Tamaulipas to the State of Yucatan (5, 6). Previous studies have reported cases in areas considered free of the disease (7, 8) and have related this to movements of the vampire bat in search of food (8). Even though this paper is about rabies in animals in Mexico in general, emphasis is put on BPR since the cases detected in cattle represent 95% of the total cases observed.

In the last decade, cattle farming in Mexico has changed—cattle are moved to new places in accordance to the human population dynamics, who move from rural to more urbanized areas (8, 9); people need cattle as a source of food. All these changes are an invitation for the vampire bat to also move searching for food (8). *D. rotundus* roost near herds of cattle and feed repeatedly, frequently in the same animal (10). Some animals may have up to four bats feeding at the same time, and 12 bites in a single night have been observed in areas where the vampire population is high (11).

In Mexico, as in some other countries, it is estimated that for every case of rabies reported in cattle, there are 10 that are not (12, 13). The non-notification of suspected cases is currently a major problem for national passive surveillance, and it is estimated that the cattle mortality rate caused by this disease is four times higher than that officially reported (14). Despite reports of low incidence of rabies in *D. rotundus* reported by the National Service of Health, Safety and Agrifood Quality of Mexico (SENASICA, acronym in Spanish) (8), cases of BPR are increasing, causing severe economic losses to the country's livestock sector, especially cattle, which represents 69% of Mexico's livestock production, worth $480 million annually through the export of live cattle (7, 15–18).

Despite the efforts to control rabies transmitted by *D. rotundus* to cattle, such as vaccination of cattle and the control of vampire bats by using chemicals to reduce population size, it is still necessary to understand the complexity of how the disease is disseminated, and spatio-temporal studies are an option to gather useful information, for example, information about factors that influence the presence of cases in areas previously free of rabies. Studies based on multivariate geostatistical methods in Mexico have partially explained the year-to-year occurrence of cases in cattle in endemic areas, and areas where it has been reported for the first time (7, 19). It has been found that environmental variables indirectly affect the ecological behavior of *D. rotundus*; however, these studies have used small databases coming from small geographic areas. It is still unclear whether all cases caused by vampire bats in cattle occur in the place where they are reported, or they were infected in endemic areas and then moved to the reported place before the disease was evident. It is not known either what environmental or anthropological factors are determining the presence of BPR in new or non-tropical areas (7, 8, 19–21).

In general, the use of time-series analysis is for characterizing patterns of behavior occurring in natural environments over time, and for analyzing fluctuations of the variable along the same time period, inferring the impact of an intervention introduced, and forecasting future responses of the variable under study (22, 23). Time series, as epidemiological models, reflect more accurately the dynamics of the epidemiological behavior of a disease despite under-reporting or over-reporting of cases, as occurs in the systematization of data on BPR (24, 25). Therefore, the purpose of this study was to perform a descriptive analysis of the distribution of cases of rabies in Mexico, and perform a time-series analysis to evaluate stationarity, and to predict the number of cases for the following year.

## Materials and Methods

### The Data

Data about cases of rabies in animal species from 2010 to 2019 were obtained from the National Service of Health, Safety and Agrifood Quality of Mexico (SENASICA, acronym in Spanish). The database includes data of all reported cases confirmed by the direct immunofluorescence diagnostic test with monoclonal antibodies against the viral nucleocapsid protein conjugated with fluorescein isothiocyanate, as indicated by Mexican Official Standard Norm (18). Epidemiological information of cases includes state, country, locality, geographic coordinates and meters above sea level (masl), date of report (month and year), information about animal (species, sex, and age), and type of antigenic variant of the rabies virus associated with the hematophagous bats *D. rotundus* (AgV3, AgV5, and AgV11), foxes (AgV7), and skunks (AgV8).

### Statistical Analysis

A descriptive analysis was performed for all variables in the data set using frequency tables and graphics for the qualitative variables, and descriptive statistics such as means, SD, and 95% CIs for the quantitative variables. All statistical analyses were performed in SPSS 22.0, released in 2013 (26).

### Spatial Analysis

Geographic coordinates for most of the cases were in the data set. Coordinates missing in the data set were obtained using Google Earth. Geographic coordinates were used to elaborate maps and identify areas with probability of cases. Coordinates were first transformed from sexagesimal units to coordinates from the universal transverse Mercator (UTM), and then to decimal units. Mappings of distribution of cases and cases per year and species were performed using ArcMap version 10.1 (27). Prediction of cases was performed with the regionalized variable “number of cases”, using ordinary kriging from the geostatistical analyst extension of ArcMap v. 10 (27). The number of cases was aggregated by municipality, for a smoothing process and to reduce the random error. Ordinary kriging is a linear-weighted interpolation method, whose weight not only depends on distance but also on the direction and orientation of the neighboring data to unsampled locations. This method assumes that data collected from a given population are spatially correlated (28, 29).

### Time-Series Analysis

An important feature of time-series analysis is that any variable measured over time is potentially influenced by previous observations (autocorrelation). To take advantage of these relationships, time-series models use previous observations as the basis for predicting future behavior. This is the essential difference between time-series analysis and traditional statistical tests for measuring change, such as regression analysis, which rely on variation in independent variables to explain changes in the outcome (30). In this study, for the time-series analysis, the total number of BPR cases was included to describe the distribution by year, and check for seasonality using an additive model. To evaluate the time-series model and to predict the number of cases in the following years, the dataset was divided into two parts, the model was run with data from 2010 to 2017, and tested with data from 2018 to 2019; prediction was made for the year 2018. All statistical analyses were performed in SPSS v.22.

## Results

The total number of rabies cases registered during the interest period from 2010 to 2019 was 3,469. Ninety-five percent of the cases occurred in domestic animals (cattle, horse, sheep, and goat); from these, 89.1% occurred in cattle (*n* = 3,091), the rest occurred in wildlife, 5% (*n* = 137), including the vampire bats *D. rotundus*, cervids, skunks, insectivorous bats, foxes, buffaloes, and coatis; 17 cases had no record about species affected (Table 1). Seventeen States reported at least 50 cases in different animal species. The cases of rabies in cattle are localized mainly in endemic States; however, some others like Sonora, Nuevo Leon, Baja California, Chihuahua, and Durango are not considered tropical or rabies endemic states but have nonetheless reported considerable number of cases (Table 2). Seventy-five percent of the cases occurred under 1,000 masl, 21.4% between 1,001 and 2,000 masl, and only 3.3% above 2,000 masl.

**Table 1:** Frequency of the number of cases of rabies in different livestock and wildlife species in Mexico, 2010–2019.

**Species**	**Number of cases**	**%**
Cattle	3,091	89.1
Horse	150	4.3
Vampire bat	109	3.1
Sheep	53	1.5
Goat	20	0.6
Unknown	17	0.5
Cervid	9	0.3
Skunk	7	0.2
Insectivorous bat (3), fox (4), buffalo (2), coati (3), pig (1)	13	0.4
**Total**	**3,469**	**100**

*The bold value 3,469 means the total number of cases and value 100 is the total percentage of the frequency of cases*.

**Table 2:** Total number of cases of rabies by state in the different livestock and wildlife species in Mexico 2010–2019.

**State**	**Number of cases**	**%**
Veracruz	491	14.2
San Luis Potosi	330	9.5
Yucatan	311	9.0
Chiapas	308	8.9
Tabasco	291	8.4
Hidalgo	252	7.3
Puebla	198	5.7
Tamaulipas	193	5.6
Nayarit	187	5.4
Guerrero	159	4.6
Jalisco	107	3.1
Quintana Roo	97	2.8
Campeche	90	2.6
Oaxaca	76	2.2
Michoacan	55	1.6
Colima	53	1.5
Queretaro	51	1.5
Zac (46), State of Mexico (33), Son (32), Mor (28), Sin (22)(24), Gto (17), BCS (10), NL (5), BC (3), Chih (3), Dgo(3), Cd. Mex. (1), Nay (1)	220	6.3
**Total**	**3,469**	**100**

*Number of cases corresponding to Zacatecas (Zac), State of Mexico, Sonora (Son), Morelos (Mor), Sinaloa (Sin), Guanajuato (Gto), Baja California Sur (BCS), Nuevo Leon (NL), Baja California (BC), Chihuahua (Chih), Durango (Dgo), Ciudad de Mexico (Cd. Mex.), and Nayarit (Nay) are shown in parentheses. The bold value 3,469 means the total number of cases and value 100 is the total percentage of the frequency of cases*.

**Figure 1:**
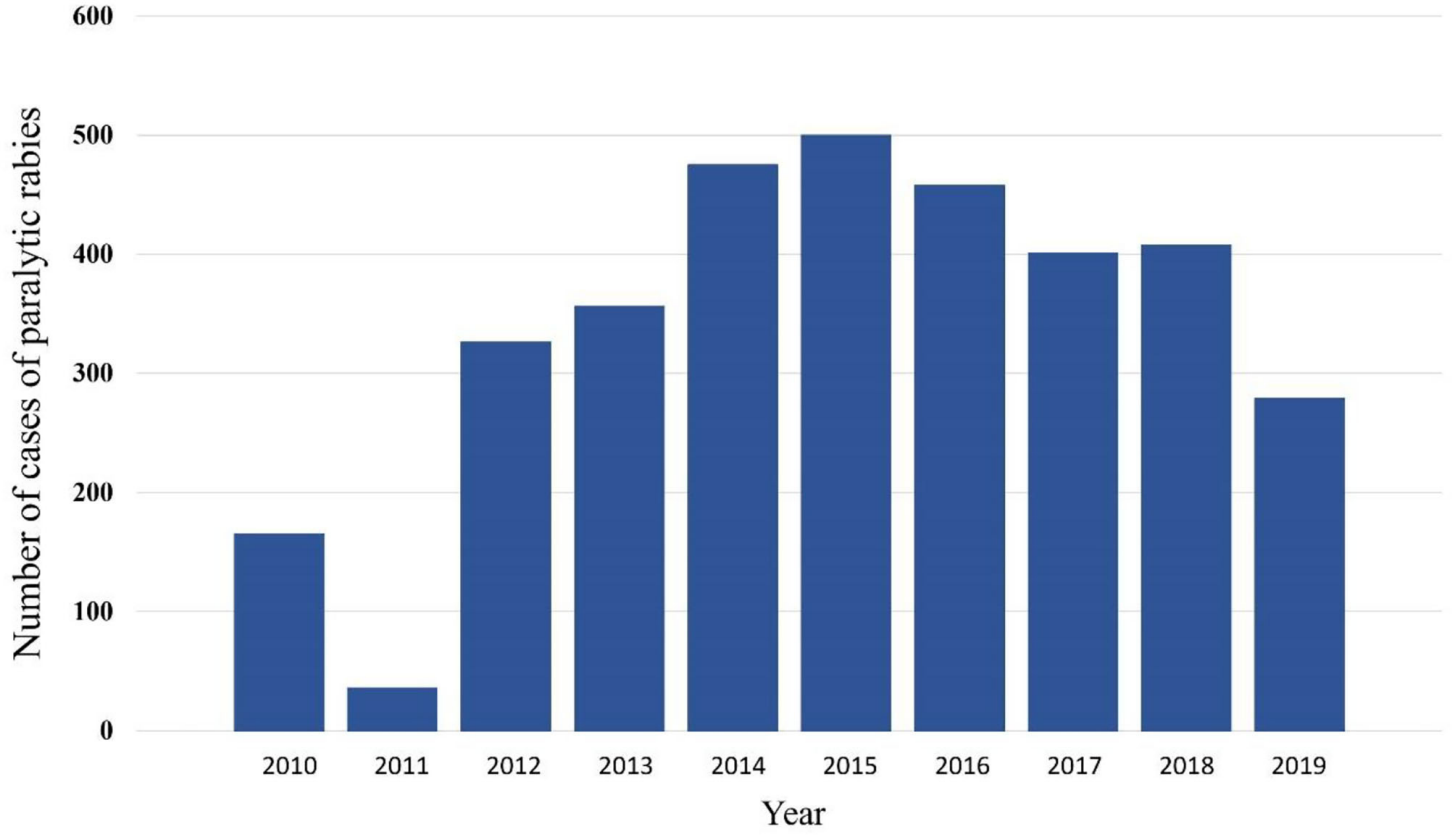
Distribution of cases of rabies in different animal species in Mexico per year, 2010–2019.

Not all cases had data about the antigenic variant of the virus. From the 194 cases that did, 76 had variant AgV11, 87 the AgV5, and 10 the AgV3, all of them associated with vampire bats. Other variants were AgV7 (two cases) associated with foxes and AgV8 (17 cases) associated with skunks. It was observed that in the state of Veracruz, the three antigenic variants associated with *D. rotundus* and the variant associated with skunks were present (Table 3). Table 4 shows the number of cases per month, the highest number of cases were reported in January, February, and March, with 360, 380, and 350 cases, respectively. The lowest frequency of cases occurred in June, July, November, and December. There was an increasing trend of cases from 2010 to 2015, and then a decreasing trend from there on. The lowest number of cases was observed in 2011 (Figure 1).

**Table 3:** Number of cases of rabies in different animal species per year, state, municipaliy, and type of antigenic variant in Mexico.

**Year**	**State**	**Number of cases**	**Municipality**	**AgV3**	**AgV5**	**AgV7**	**AgV8**	**AgV11**
2012								
	Veracruz	16	7	–	–	–	–	16
	Guerrero	2	2	–	–	–	2	–
	Puebla	6	4	–	–	–	–	6
2013								
	Veracruz	27	18	–	–	–	–	27
	Guerrero	2	2	1	–	–	1	–
	Baja California	2	1	–	–	–	1	1
	Colima	1	1	–	–	–	–	1
	Oaxaca	2	2	–	–	–	–	2
2014								
	Veracruz	42	30	1	17	–	2	22
	Colima	6	3	–	1	–	5	–
	Hidalgo	1	1	–	–	–	–	1
	Michoacán	2	2	–	–	–	2	–
	Zacatecas	2		–	–	–	2	–
	Guerrero	2	2	1	–	–	1	–
2015								
	Veracruz	65	1	–	65	–	–	–
	Morelos	2	1	–	2	–	–	–
	Michoacán	1	1	–	1	–	–	–
	Guerrero	1	1	–	1	–	–	–
2016								
	Veracruz	1	1	1	–	–	–	–
	Campeche	1	1	1	–	–	–	–
	Puebla	1	1	–	–	1	–	–
	San Luis Potosi	1	1	1	–	–	–	–
	Hidalgo	1	1	–	–	–	1	–
	Sonora	4	1	3	–	1	–	–
2017								
	Sonora	1	1	1	–	–	–	–

*Antigenic variants associated to D. rotundus: AgV3, AgV5, AgV11. Antigenic variant associated to foxes: VAgV7. Antigenic variants associated to skunks: AgV8. Of the 194 cases that reported type of antigenic variant, two were atypical*.

**Table 4:** Frequency of the number of cases of rabies in different livestock and wildlife species by month for the period 2010–2019 in Mexico.

**Month**	**Number of cases**	**%**
January	360	10.4
February	380	11.0
March	350	10.1
April	246	7.1
May	282	8.1
June	236	6.8
July	220	6.3
August	322	9.3
September	282	8.1
October	296	8.5
November	211	6.1
December	221	6.4
Total	3,469	100

**Figure 2:**
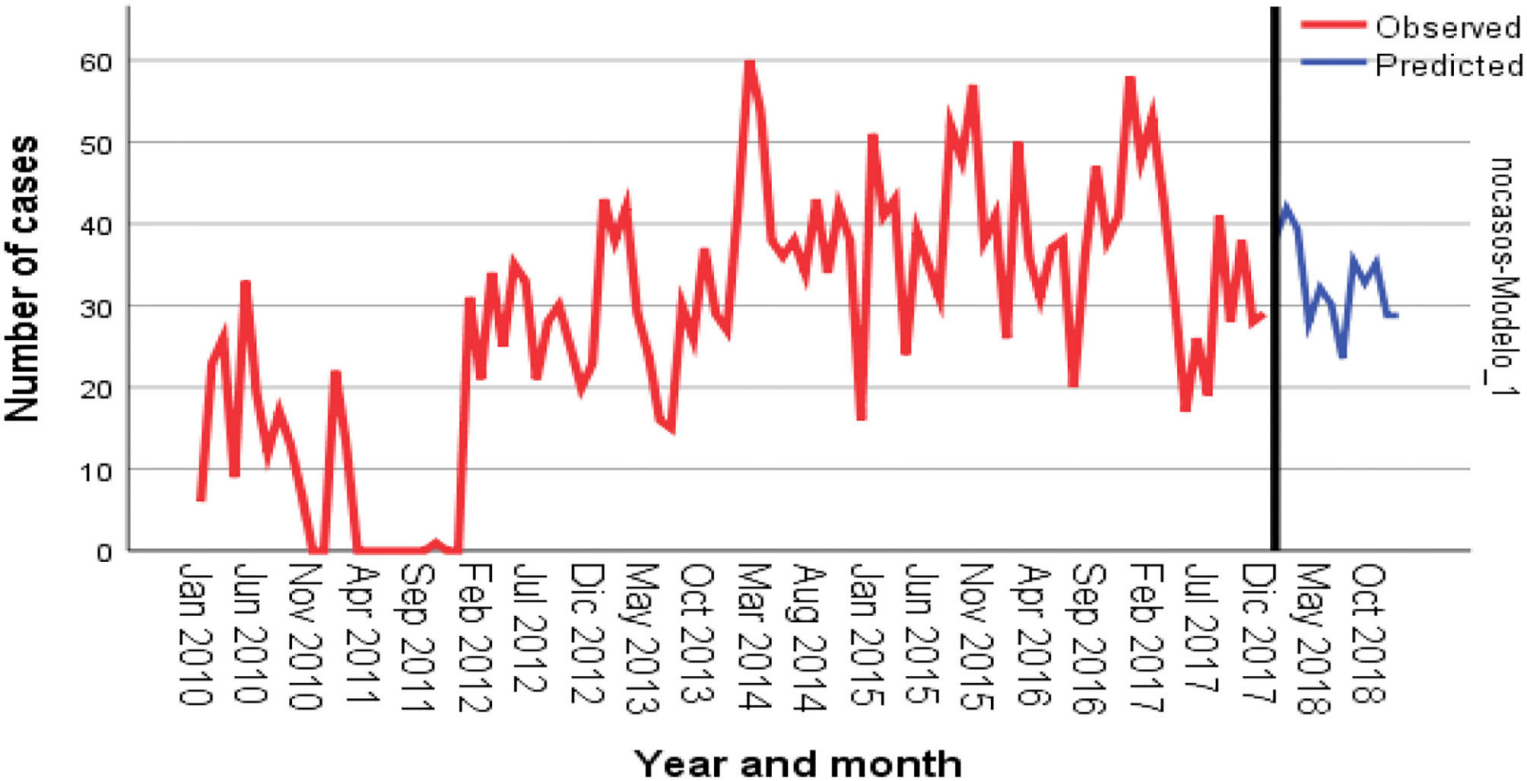
Time-series analysis and prediction of number of cases in different animal species in Mexico. Red line plots the observed values; blue line, the adjusted and the predicted values. Forecasting was performed for the year 2018.

Figure 2 shows the time-series frequency and the predicted number of cases. A slight seasonal effect was observed at the beginning of each year, from January to March. The real and the predicted values of cases in all species for the year 2018 obtained in the time-series analysis used for predicting data for the years 2010 to 2017 are shown in Table 5. The model showed good predicting value; the Pearson correlation coefficient R^2^ was 0.705.

**Table 5:** Predicted and real values of the number of observed rabies cases in different animal species in Mexico 2010–2019.

**Year**	**Month**	**Real values**	**Predicted values**	**95% CI**	
				**Low**	**Up**
2018	January	62	38	21	55
2018	February	48	42	23	61
2018	March	44	39	19	60
2018	April	38	28	6	50
2018	May	43	32	8	56
2018	June	22	30	5	56
2018	July	46	24	−3	50
2018	August	49	35	7	63
2018	September	30	33	3	62
2018	October	24	35	5	66
2018	November	0	29	−3	60
2018	December	2	29	−4	62

*Trial period: 2010–2017. Prediction period, 2018*.

**Figure 3:**
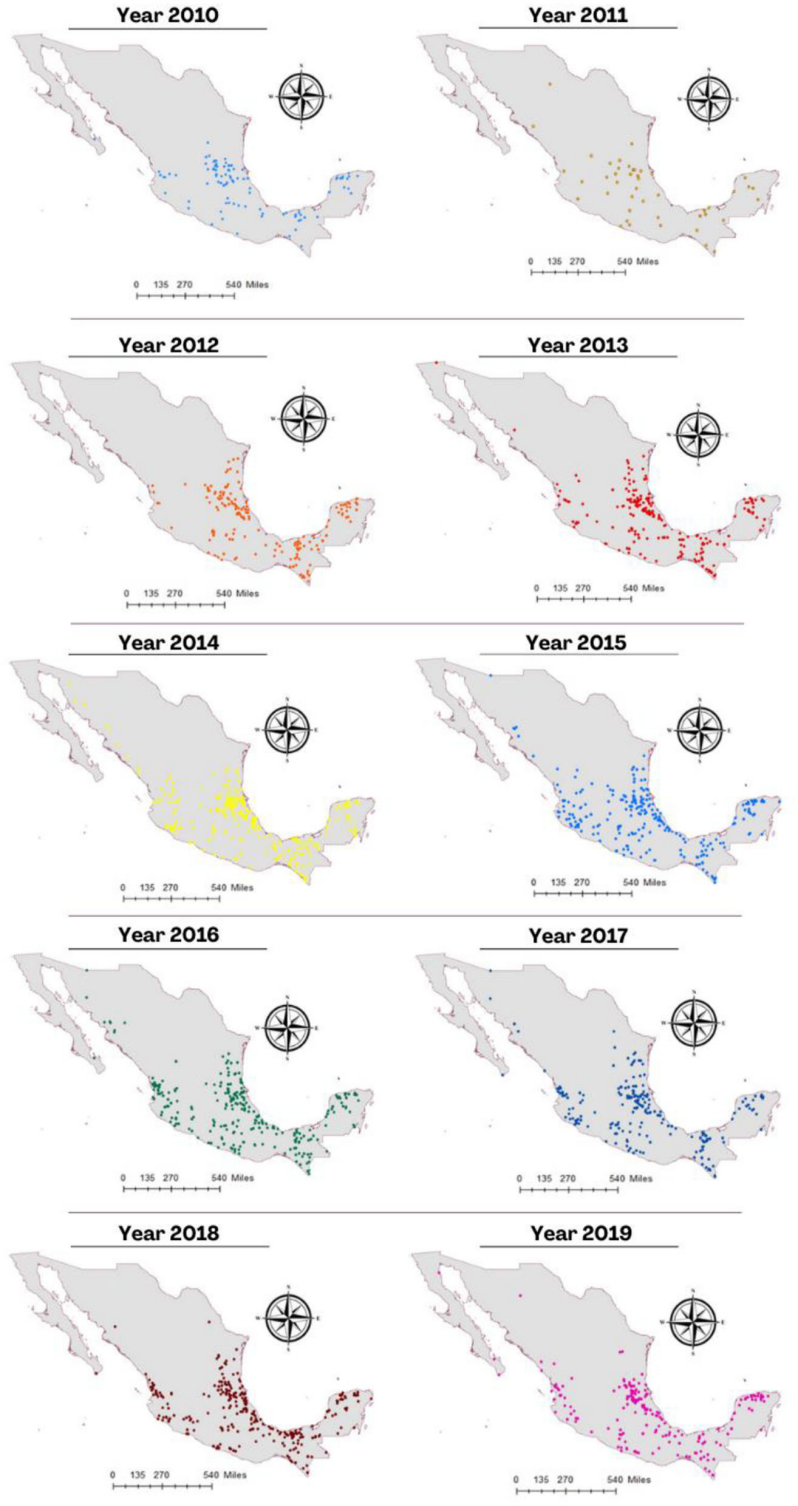
Distribution of cases of rabies in different species animals per year during the period 2010–2019.

**Figure 4:**
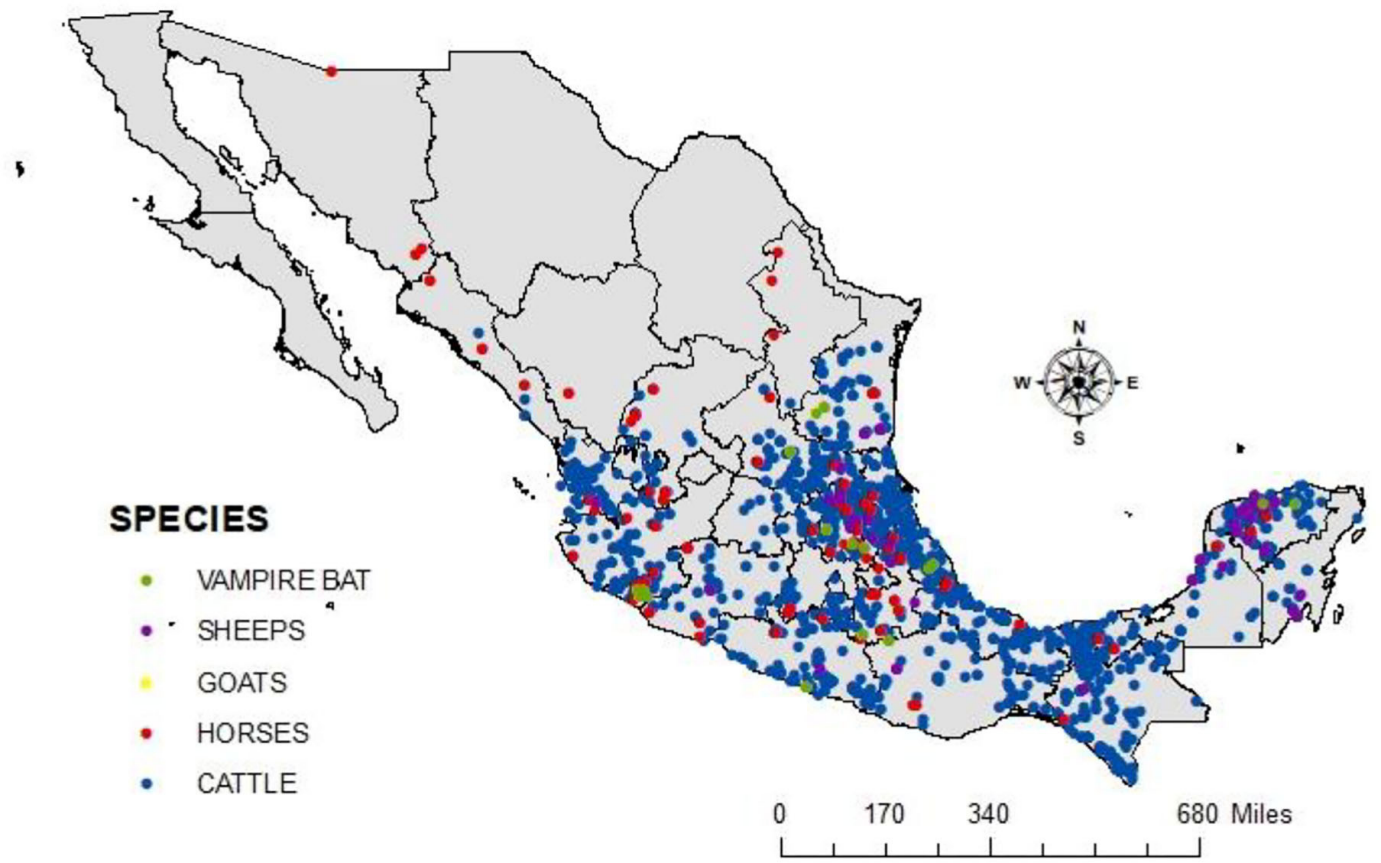
The spatial distribution of cases in livestock species and vampire bats from 2010 to 2019. Rabies cases in cattle are shown in blue, horses in red, vampire bats in green, goats in yellow, and sheep in purple.

Figure 3 shows the spatial distribution of cases of rabies in different animal species per year during 2010–2019. There is a clear pattern of dissemination to areas where the disease had not been previously reported, such as the center and north of Mexico. The spatial distribution of cases in all livestock species and vampire bats in the same period is shown in Figure 4. It is observed that most cases corresponding to cattle occurred in the coasts the Pacific and the Gulf of Mexico. An area with a high number of cases is observed in the junction of the States of Veracruz, Tamaulipas, Guanajuato, Queretaro, and San Luis Potosi. This area called “The Huasteca” has traditionally been known as an endemic region that needs permanent attention and probably one of the sources of infection for other regions. Surprisingly, cases are observed in the States of Sonora and Chihuahua, which are dry and arid, with an environment hostile for the vampire bat.

**Figure 5:**
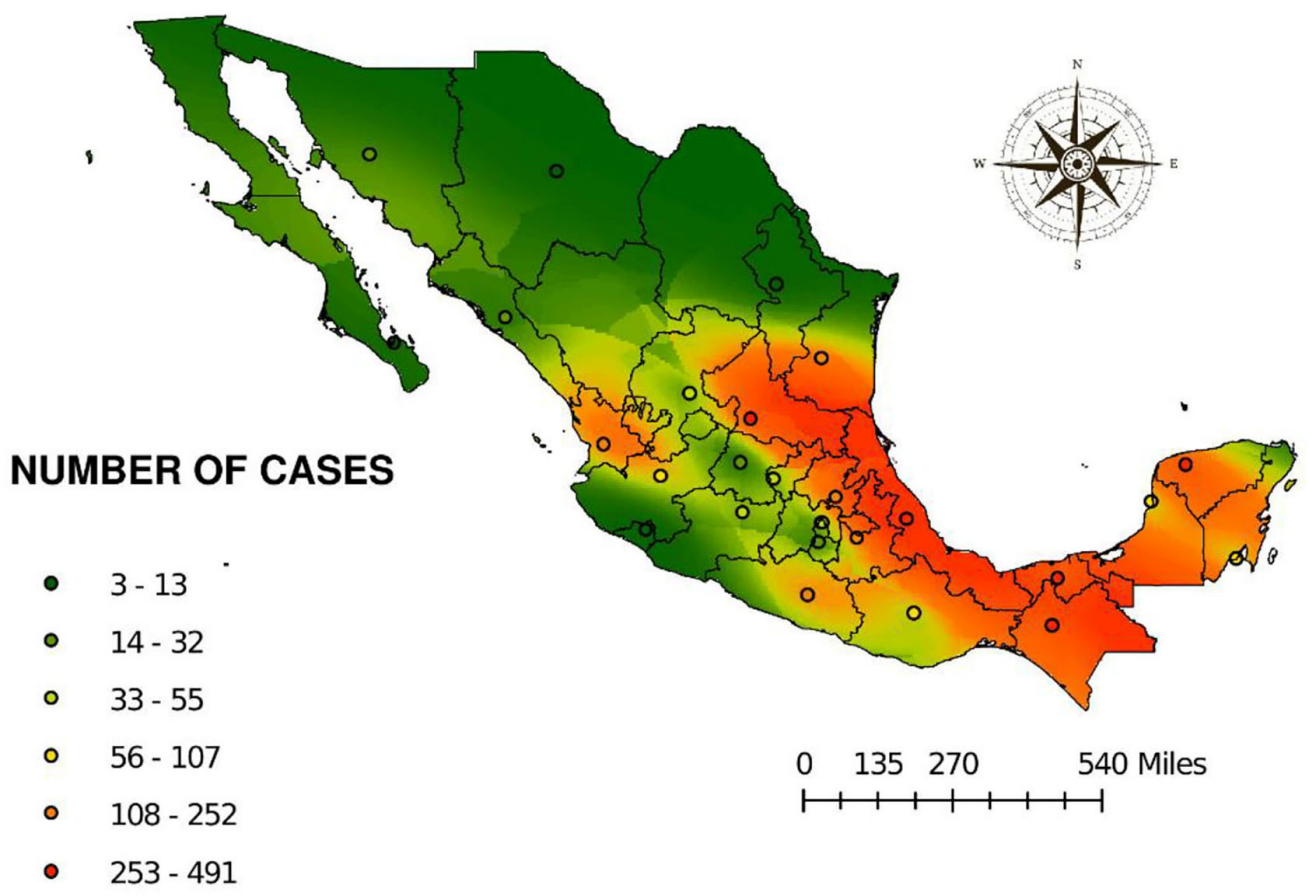
Prediction of risk to cases of rabies in different species from 2010 to 2019 by ordinary kriging.

Figure 5 shows the risk map obtained by ordinary kriging. This map is based on the regionalized variable of number of cases. The spherical semivariogram model was as follows: 0 ^*^ Spherical ^*^ [4.35] + 118.47 ^*^ Nugget with a value for the standardized mean of −0.36 and the standard error of 1.15 indicating an acceptable model. Prediction clearly shows high risk in the areas located in the coast of the Gulf of Mexico, involving the States of Tamaulipas, Veracruz, Tabasco, Chiapas, Yucatan, Campeche, and Quintana Roo. In the Pacific coast, the states with higher risk are Oaxaca, Guerrero, and Nayarit. As expected, the high-risk areas correspond to the typical distribution of the hematophagous bat *D. rotundus*. However, a high-risk tendency was observed in the regions of Huasteca Potosina, Huasteca Hidalguense, the north of the country over the coast line throughout Tamaulipas, and the center of the country in the region known as the Bajio.

## Discussion

Our results show that the highest frequency of cases occurred in cattle in both coasts of Mexico, the Pacific and the Gulf of Mexico coast, with an important contribution of the States of San Luis Potosi. These results agree with previous reports (31), who reported the highest number of cases in Veracruz, San Luis Potosi, Yucatan, Tabasco, and Chiapas for data from 2007 to 2015. It agrees also with a recent study in central Mexico, where the State of San Luis Potosi had the highest number of cases in central Mexico (19).

Our study shows that in recent years, cases of rabies have been reported in areas where the disease had not been observed before, such as some regions of the States of Guanajuato and Queretaro, where the climatic conditions are not suitable for the vampire bat *D. rotundus* (19). However, it has been hypothesized that non-environmental, anthropogenic factors have a role in the disease distribution, for example, livestock population density, changes in agricultural activities, new buildings and bridges, and some others (8, 32). Areas with topography with caves also favor the presence of the vampire bat (33). It has been documented that the vampire bat can adapt to different ecosystems as far as it has feeding sources, where livestock might have an important role (7, 34).

The highest frequency of cases was observed in cattle, horses, sheep, and goat, species traditionally affected by *D. rotundus* (35). Livestock management may play a role in this; in some geographic areas of Mexico, it is common to congregate cattle and other domestic animals during the rainy season in corrals close to the family house (21, 36), and this might be an opportunity for *D. rotundus* to attack when roosting near herds of cattle and feed repeatedly and frequently in the same animal (10). Some animals may have four or more bats feeding at the same time and have 12 bites in a single night (11).

Only five types of the antigenic variant of the RABV were observed, which correspond to wild animals, three to *D. rotundus*. The distribution of vampire bat populations matches the distribution of cases in domestic and wildlife animals, where variants AgV11, AgV3, and AgV5 associated with *D. rotundus* have been reported (37). However, the presence of cases in wild species in non-endemic areas of Mexico is remarkable; nevertheless, the variant V8 in skunks in Chihuahua has been previously reported (38).

The highest frequency of cases occurs between February and March each year. This might be due to management practices of cattle during that period. Between November and April, grass is scarce in some tropical regions and farmers gather the animals in corrals to provide silage and hay; this may be used for the vampire to obtain food easily. It might also be explained by the reproductive behavior of bats—the reproductive season of *D. rotundus* occurs during this time, and juvenile vampires require constant feeding to complete their development, meet the energy demands necessary for reproduction, and, in the case of males, to move to other colonies in search of females (39–42). Considering that the gestation period in bats is 205 days, the female bats pregnant in March or April will give birth in September or October, so it is possible that during the months of May, June, July, and August, the cases of rabies decrease a little. This is because near the calving date, the flight hours decrease considerably due to the weight and bulging of the abdominal region, which make the female expend more energy to find food (11, 43).

The annual frequency of observed cases of rabies shows an increasing pattern from 2010 to 2015, and then decreasing from 2016 to 2019. This pattern has been observed in other countries in Latin America (21). The increase in the number of cases in livestock could be more likely related to official policies and provision of economic resources to the national rabies control program rather than to a real natural phenomenon.

Underreporting is another issue. The main critical point for surveillance lies in the systematization of case reporting. For example, in the endemic area of southeastern Mexico, 95% of farmers affected with cases of rabies in livestock do not notify to competent authorities about the disease. This may also occur in other geographic regions (21). This is reflected in the fact that the municipalities who send a high number of samples to the laboratory are also the ones with the highest number of detected cases (21, 34, 44).

Results from the time-series analysis reflected a seasonal pattern of cases. This seasonality could be associated with the variability of monthly temperature in some geographic areas. Something similar has been observed in Brazil and Colombia where the higher number of cases in livestock reached the peak in the months of January, April, and October (32, 45). In Mexico, the months of December to March are the coldest months of the year; therefore, it is possible that cattle are calm and close to each other to avoid the cold weather, making easier for the bats to attack. It has been reported that in some regions, the population density of *D. rotundus* in refuges is higher during the spring (46), which could be associated with more cases at this time of the year.

Figure 5 shows the risk for the presence of cases of rabies. It can be observed that there is a tendency of risk toward not endemic or areas free of the disease, suggesting that due to the lack of policies to prevent the spread of the disease, cases could be detected in those areas in the near future, especially if those areas offer microclimates suitable for the vampire bat *D. rotundus* (7, 21, 47).

## Conclusions

This study confirms that rabies in domestic and wildlife species is endemic in tropical and subtropical areas in both the Pacific and the Gulf of Mexico coasts, and that its distribution is clearly associated with the distribution of the reservoir vampire bat *D. rotundus*. However, it has been shown also that cases occur in areas with environmental conditions not appropriate for the reservoir bat, suggesting that the dissemination of the disease depends on factors other than just environmental, and that if actions are not taken to stop the spread, such as well-planned vaccination strategies and bat population control, considering time and place, the problem may grow.

## Data Availability Statement

The raw data collection is from the National Epidemiological Surveillance System of Mexico. Requests to access the datasets should be directed to Isabel Bárcenas isabel.barcenas@uaq.mx.

## Author Contributions

RO-S: investigation, methodology, formal analysis, and writing—original draft. IB-R: conceptualization, statistical model generation, supervision, and writing—review and editing. GC-A: review and editing. JL-C: statistical model generation, review, and editing. R-AE: review and editing. YC-M: review and editing. SG-R: review and editing. BC-G: providing the information on paralytic rabies cases during the 2010–2019 period in Mexico. FM-S: formal analysis and writing—review and editing. All authors contributed to the article and approved the submitted version.

## Conflict of Interest

The authors declare that the research was conducted in the absence of any commercial or financial relationships that could be construed as a potential conflict of interest.

## Publisher's Note

All claims expressed in this article are solely those of the authors and do not necessarily represent those of their affiliated organizations, or those of the publisher, the editors and the reviewers. Any product that may be evaluated in this article, or claim that may be made by its manufacturer, is not guaranteed or endorsed by the publisher.

## References

[1] Mochizuki N Kobayashi Y Sato G Hirano S Itou T ITO FH. Determination and molecular analysis of the complete genome sequence of two wild-type rabies viruses isolated from a haematophagous bat and a frugivorous bat in Brazil. J Vet Sci. (2011) 1101250445–1101250445. 10.1292/jvms.10-0238 21301181

[2] De La Rosa ES Kotait I Barbosa TF Carrieri ML Brandão PE Pinheiro AS. Bat-transmitted human rabies outbreaks, Brazilian Amazon. Emerg Infect Dis. (2006) 12:1197. 10.3201/eid1208.050929 16965697 PMC3291204

[3] Loza-Rubio E Rojas-Anaya E López J Olivera-Flores MT Gómez-Lim M Tapia-Pérez G. Induction of a protective immune response to rabies virus in sheep after oral immunization with transgenic maize, expressing the rabies virus glycoprotein. Vaccine. (2012) 30:5551–6. 10.1016/j.vaccine.2012.06.039 22749836

[4] PAHO. (2019). Available onlne at: https://www.paho.org/es/noticias/16-12-2019-mexico-recibe-certificado-validacion-eliminacion-rabia-humana-transmitida-por

[5] Lee S Thiem D Anh D Duong N Lee M Grace D. Geographical and temporal patterns of rabies post exposure prophylaxis (PEP) incidence in humans in the Mekong River Delta and Southeast Central Coast regions in Vietnam from 2005 to 2015. PLoS. (2012) 13:e0194943. 10.1371/journal.pone.0194943 29634746 PMC5892892

[6] Peel DS Mathews KH Jr Johnson RJ. Trade, the Expanding Mexican Beef Industry, Feedlot Stocker Cattle Production in Mexico. Washington, DC: U.S. Department of Agriculture, Economic Research Service (2011). Available online at: http://www.ers.usda.gov/Publications/LP/2011/08Aug/LDPM20601/ldpm2060.pdf

[7] Bárcenas-Reyes I Nieves-Martínez DP Cuador-Gil JQ Loza-Rubio E González-Ruiz S Cantó-Alarcón GJ. Spatiotemporal analysis of rabies in cattle in central Mexico. Geospat Health. (2019) 14:805. 10.4081/gh.2019.805 31724374

[8] Romero-Barrera CE Osorio-Rodriguez AN Juárez-Agis A. Distribución. abundancia, control y registros de casos de murciélagos vampiro, *Desmodus rotundus* (geoffroy) e, infectados de rabia en ambientes pecuarios de guerrero, Mexico: population control of the vampire bat. Acta Agrícola y Pecuaria. (2021) 7. 10.30973/aap/2021.7.0071005

[9] SIAP. Población ganadera. (2020). Available onlne at: https://www.gob.mx/siap/documentos/poblacion-ganadera-136762.

[10] Turner C. The Vampire Bat. Baltimore, Md: Johns Hopkins University Press. (1975).

[11] Greenhall M Joermann G Schmidt U. The use of precipitin test to determine the host preferences of the vampire bats *Desmodus rotundu*s and *Diaemus youngi*. Bijd Dierkunde. (1983) 40:36–9. 10.1163/26660644-04001011

[12] Fornes A Lord RD Kuns ML Largui OP Fuenzalida E Lazara L. Control of bovine rabies through vampire bats control. J Wildl Dis. (1974) 10:310–6. 10.7589/0090-3558-10.4.310 4436917

[13] Escobar Cifuentes E. Rabies transmitted by vampires. Biomédica. (2004) 24:231–6. 10.7705/biomedica.v24i3.1268 15551874

[14] Benavides J Valderrama W Streicker D. Spatial expansions and travelling waves of rabies in vampire bats. Proc R Coc B. (2017) 283:20160328. 10.1098/rspb.2016.0328

[15] Leos-Rodríguez JA Serrano-Páez A Salas-González JM Ramírez-Moreno PP Sagarnaga-Villegas M. Caracterización de ganaderos y unidades de producción pecuaria beneficiarios del programa de estímulos a la productividad ganadera (PROGAN) en México. Agricultura, sociedad y desarrollo. (2008) 5:213–30.

[16] SADER. (2018). Available onlne at: https://www.gob.mx/pronabive/articulos/la-importancia-de-controlar-la-rabia-paralitica-bovina?idiom=es

[17] SADER. Dirección de Campañas Zoosanitarias. (2019). Available onlne at: https://www.gob.mx/senasica/acciones-y-programas/programas-y-campanas-zoosanitarias (consultado 5/03/2020).

[18] NOM-067-ZOO-2007. Norma Oficial Mexicana, Campaña nacional para la prevención y control de la rabia en bovinos y especies ganaderas. Diario Oficial de la Federación, 20 de mayo de 2011. Available online at: https://www.gob.mx/cms/uploads/attachment/file/203509/NOM-067-ZOO-2207_20may11_Ori.pdf

[19] Bárcenas-Reyes I Loza-Rubio E Zendejas-Martínez H Luna-Soria H Cantó-Alarcón GJ Milián-Suazo F. Comportamiento epidemiológico de la rabia paralítica bovina en la región central de Mexico, 2001-2013. RPSP. (2015) 38:396–402. 26837525

[20] Bárcenas-Reyes I Loza-Rubio E Cantó-Alarcón GJ Luna-Cozar J Enríquez-Vázquez A Barrón-Rodríguez RJ. Whole genome sequence phylogenetic analysis of four Mexican rabies viruses isolated from cattle. Res Vet Sci. (2017) 113:21–4. 10.1016/j.rvsc.2017.08.004 28818750

[21] Mendoza-Sáenz VH Navarrete-Gutiérrez DA Jiménez-Ferrer G Kraker-Castañeda C Saldaña-Vázquez RA. Abundance of the common vampire bat and feeding prevalence on cattle along a gradient of landscape disturbance in southeastern Mexico. Mammal Research. (2021) 66:481–95. 10.1007/s13364-021-00572-9

[22] Chatfield C. The Analysis of Time Series, 5th ed. London: Chapman and Hall. (1996).

[23] Makridakis SG Wheelwright SC Hyndman RJ. Forecasting: Methods and Applications, 3rd ed. New York: John Wiley and Sons. (1998).

[24] Betancourt Betancourt J Santana Brito H Ortiz Hernández E Rodríguez Socarrás N. Caracterización y análisis de series de tiempo de enfermedades respiratorias agudas en la provincia de Camagüey. AMC. (2009) 13: ISSN 1025–0255.

[25] Rocha F Ulloa-Stanojlovic FM Rabaquim VCV Fadil P Pompei JC Brandão PE. Relations between topography, feeding sites, and foraging behavior of the vampire bat, Desmodus rotundus. J Mammal. (2020) 101:164–71. 10.1093/jmammal/gyz177

[26] SPSS IBM Corp. IBM SPSS Statistics for windows, V 22.0. Armonk, NY: IBM Corp. (2013).

[27] ESRI. ArcGIS Desktop: Release 10. Redlands: Environmental Systems Research Institute. (2011).

[28] Matheron G. La teoría de las variables regionalizadas y sus aplicaciones. En: Los Cuadernos del Centro de Morfología Matemática de Fontainebleau. Fascículo 5. París: Centro de Geoestadística de la Escuela de Minas de París. (1970). p. 125.

[29] Caro A. (2012). Modelización Geoestadística para la predicción de actividad de 137Cs en el suelo. Doctoral dissertation, Tesis Doctoral. Ed. Madrid: Universidad Autónoma de.

[30] Linden A Adams JL Roberts N. Evaluating disease management program effectiveness: an introduction to time-series analysis. Dis Manag. (2003) 6:243–55. 10.1089/109350703322682559 14736348

[31] Zarza H Martínez-Meyer E Suzán G Ceballos G. Geographic distribution of Desmodus rotundus in Mexico under current and future climate change scenarios: Implications for bovine paralytic rabies infection. Vet Mex. (2017) 3:1–16. 10.21753/vmoa.4.3.390

[32] Cárdenas Contreras ZL. Análisis espacio temporal de la rabia bovina de origen silvestre en. Colombia. (2017).

[33] Jiménez-Guzmán A Zúñiga R. Nuevos registros de mamíferos para Nuevo León, Mexico. Publ Biol. (1992) 6:189–91.

[34] Hayes MA Piaggio AJ. Assessing the potential impacts of a changing climate on the distribution of a rabies virus vector. PLoS One. (2018) 13: e0192887. 10.1371/journal.pone.0192887 29466401 PMC5821341

[35] Mayen F. Haematophagous bats in Brazil, their role in rabies transmission, impact on public health, livestock industry and alternatives to an indiscriminate reduction of bat population. J Vet Med, Series B. (2003) 50:469–72. 10.1046/j.1439-0450.2003.00713.x 14720182

[36] Lanzagorta-Valencia K Fernández-Méndez JI Medellín RA Rodas-Martínez AZ Ávila-Flores R. Landscape cattle management attributes associated with the incidence of Desmodus rotundus attacks on cattle Características del paisaje y de manejo ganadero asociadas a la incidencia de ataques al ganado bovino por Desmodus rotundus. Landscape. (2020) 7:1–10. 10.19136/era.a7n1.2164

[37] Aréchiga-Ceballos N Almazán-Marín C Aguilar-Setién Á. Transmisión vertical del virus de la rabia cría-madre, fenómeno que podría mantener al virus en especies reservorios de vida silvestre. Gac Med Mex. (2019) 155:249–53. 10.24875/GMM.19005013 31219468

[38] Jaramillo-Reyna E Almazán-Marín C de la O-Cavazos ME Valdéz-Leal R Bañuelos-Álvarez AH Zúñiga-Ramos MA. Public veterinary medicine: public health rabies virus variants identified in Nuevo Leon State, Mexico, from 2008 to 2015. JAVMA. (2020) 256: 438–443. 10.2460/javma.256.4.438 31999515

[39] Lord RD Delpietro H Fuezauda E De Diaz AMO Lazaro L. Presence of rabies neutralizing antibodies in wild carnivores following an outbreak of bovine rabies. J Wildl Dis. (1975) 11:210–3. 10.7589/0090-3558-11.2.210 1142554

[40] Safi K. Social bats: the males' perspective. J Mammal. (2008) 89:1342–50. 10.1644/08-MAMM-S-058.1

[41] Blackwood JC Streicker DG Altizer S Rohani P. Resolving the roles of immunity, pathogenesis, and immigration for rabies persistence in vampire bats. PNAS. (2013) 110:20837–42. 10.1073/pnas.1308817110 24297874 PMC3870737

[42] Johnson N Aréchiga-Ceballos N Aguilar-Setien A. Vampire bat rabies: ecology, epidemiology and control. Viruses. (2014) 6:1911–28. 10.3390/v6051911 24784570 PMC4036541

[43] Kobayashi M. Atributos Anatómicos del Músculo Cuádriceps Femoral Responsable de la Limitada Capacidad de Arrastre en el Murciélago Grande de Herradura (Rhinolophus ferrumequinum). J Morphol. (2018) 36:69–73.

[44] Correa K Iamamoto K Miyuki K Mori E Estevez A Achkar S. Murciélagos hematófagos como reservorios de la rabia. Rev Peru Med Exp Salud Publica. (2014) 31:302–9. 10.17843/rpmesp.2014.312.51 25123871

[45] Tolosa-Quintero NJ Loboa-Rodríguez NJ Gutiérrez-Lesmes OA Gongora-Orjuela A. Indicador compuesto en salud: riesgo de transmisión del virus de la rabia. Rev Esp Salud. (2020) 20:752–8. 10.15446/rsap.v20n6.74695 33206901

[46] Ortiz Badillo RM. Diversidad de murciélagos en un gradiente altitudinal en el estado de Nuevo León, Mexico. Doctoral dissertation. Universidad Autónoma de Nuevo León. (2015).

[47] Benavides JA Valderrama W Recuenco S Uieda W Suzán G Avila-Flores R. Defining new pathways to manage the ongoing emergence of bat rabies in Latin America. Viruses. (2020) 12:1002. 10.3390/v12091002 32911766 PMC7551776

